# Is It a New Approach for Treating Senile Hypertension with Kidney-Tonifying Chinese Herbal Formula? A Systematic Review of Randomized Controlled Trials

**DOI:** 10.1155/2014/473038

**Published:** 2014-02-17

**Authors:** Jie Wang, Xingjiang Xiong, Xiaochen Yang

**Affiliations:** Department of Cardiology, Guang'anmen Hospital, China Academy of Chinese Medical Sciences, Beijing 100053, China

## Abstract

*Objective*. To assess the effect of kidney-tonifying (KT) Chinese herbal formula for senile hypertension (SH). *Methods*. A total of five databases (CENTRAL, Pubmed, CBM, CNKI, and VIP) were searched for published and unpublished randomized controlled trials (RCTs) up to November 30th, 2013. We included RCTs that confoundedly addressed the effect of KT formula in the treatment of SH. *Results*. Six studies involving 527 people were included. There was no evidence that KT formula alone had superior effects to antihypertensive drugs in systolic blood pressure (MD: −3.70 (−11.47, 4.07); *P* = 0.35) and diastolic blood pressure (MD: −3.00 (−7.26, −1.26); *P* = 0.17). However, there was evidence that KT formula combined with antihypertensive drugs was a better treatment option than antihypertensive drugs alone in systolic blood pressure (WMD: −8.69 (−12.35, −5.02); *P* < 0.00001) but no significant difference in diastolic blood pressure (WMD: −1.32 (−7.93, −5.30); *P* = 0.7). In addition, after several weeks of treatment, the level of blood lipid, endothelin, blood urea nitrogen, and creatinine decreased significantly (*P* < 0.05). *Conclusion*. Compared with antihypertensive drugs alone, KT formula combined with antihypertensive drugs may provide more benefits for patients with SH. The large-scale high-quality trials are warranted in future.

## 1. Introduction

Senile hypertension (SH), a common cardiovascular disease among senile people, refers to the condition in patients over 60 years old who have a diastolic blood pressure (DBP) ≥12 kPa (90 mmHg) and a systolic blood pressure (SBP) ≥18.6 kPa (140 mmHg) or only have isolated systolic hypertension. As the average age increases in China, SH incidence is rising yearly and up to 75% among people over 60 [1]. Among the old people, high blood pressure (BP) has been proved to directly increase cardiovascular risk about three or four times [2]. Increased SBP has been recommended to be the major criterion for diagnosis, staging, and therapeutic management of SH [3, 4]. Several lines of strong evidence support the initiative to emphasize SBP. From the perspectives of pathology, the prevalence of cardiac and vascular disease is associated with aging, increased stiffness of large arteries, increased SBP, and increased pulse pressure frequently [5]. From the perspectives of epidemiology, isolated systolic hypertension is the most common form of hypertension and is present in approximately two-thirds of hypertensive individuals > 60 years of age [6]. From the perspectives of diagnosis, classification and staging of hypertension are more precise when systolic rather than diastolic BP is used as the principal criterion [7]. Preventing the complications of hypertension, including stroke, coronary artery disease, myocardial infarction, heart failure, and kidney failure, may potentially impact not only an individual's functional status but also their ability to live independently in the community [8]. Despite the increased absolute risk for cardiovascular events associated with hypertension in the elderly, significant numbers of individuals remain untreated or inadequately treated [9]. Clinical benefits of treatment of isolated systolic hypertension include reductions in stroke, myocardial infarction, heart failure, kidney failure, and overall cardiovascular disease morbidity and mortality. For treatment, because the course of illness is long and accompanied with various degrees of visceral lesions of the heart, brain, and kidney, it has been proved by clinical experience that the blood pressure cannot be made to decrease too fast nor decrease too slow so as to prevent the severe complications which may happen after a long-term high blood pressure [10].

To treat senile hypertension, most patients need combination therapy, which means to take at least two or three drugs for achieving target BP every day and night. In addition to small dosage of such antihypertensive drugs as captopril (12.5 mg), nifedipine (10 mg), and atenolol (12.5 mg), Chinese herbal medicine (CHM) can be prescribed based on traditional Chinese medicine (TCM) differentiation of the syndromes for lowering BP, improving the blood supply of the target organs, and relieving the clinical symptoms. With the increasing prevalence of hypertension, there is a growing tendency for patients, especially for senile patients, to turn to CHM [11, 12]. Several TCM clinical and experimental studies, including a substantial number of randomized controlled trials (RCTs), have shown that CHM is effective and safe for the treatment of hypertension [13, 14]. One of the most important reasons for CHM to treat senile hypertension and reduce its complications is that TCM characterizes treatment by syndrome differentiation [15]. According to theory of The Yellow Emperor' s Inner Classic (Huáng Dì Nèi Jīng), the kidney-deficiency (includes kidney-qi deficiency, kidney-yin deficiency, and kidney-yang deficiency) begins to occur on the elderly and leads to some symptoms such as tinnitus, lumbago, and dizziness. The common ingredients of the prescription for kidney-deficiency include Lu Rong (Cornu Cervi Pantotrichum), Yin Yang Huo (Herba Epimedii), and Tu Si Zi (Semen Cuscutae) for tonifying kidney-yang.

Although treating senile hypertension with kidney-tonifying (KT) Chinese herbal formula had experienced hundreds of years and seems to have good effects in clinical practice, the evidence examining the efficacy of KT formula for senile hypertension has never been systematically summarized. Thus, we performed this systematic review to critically assess the effect and safety of KT formula for the treatment of senile hypertension.

## 2. Methods

### 2.1. Eligibility Criteria

We included randomized controlled trials that compared KT formula with placebo or antihypertensive drugs in old patients (age > 60) with essential hypertension (DBP ≥ 90 mmHg and SBP ≥ 140 mmHg) or these only have isolated systolic hypertension. The eligible comparisons include

- KT formula versus any current antihypertensive medications;
- KT formula plus any current antihypertensive medications for senile hypertension versus antihypertensive medications alone;
- KT formula versus placebo.

Our prespecified primary outcome is all-cause mortality, and secondary outcomes include blood pressure (BP), the level of blood lipids (BL), endothelin (ET), blood urea nitrogen (BUN), creatinine (Cr), and adverse events.

### 2.2. Search Strategy

Two authors (X. Xiong and X. Yang) searched online databases including the Cochrane Central Register of Controlled Trials (CENTRAL, 2013), Pubmed (1950–2013), Chinese Biomedical Literature Database (CBM, 2013), Chinese National Knowledge Infrastructure (CNKI, 2013), and Chinese Scientific Journal Database (VIP, 2013) up to November 30th, 2013. The English searching terms were used individually or combined including “senile hypertension”, “Kidney-tonifying”, “Chinese herbal formula”, “randomized controlled trial”, “controlled clinical trial”, “randomly”, “trial”, “randomised”, and “randomized”. The Chinese searching terms were used individually or combined including those for the generic name of senile hypertension *“Lao_nian_xing_gao_xue_ya”*, kidney-tonifying *“Bu_shen”*, and randomized *“sui_ ji”*. No language restriction was applied. The detailed search strategy of each database is available as supporting information.

We also searched unpublished postgraduate theses in Chinese databases. The reference lists of all relevant papers found electronically were hand-searched. Meanwhile, experts in this field and relevant pharmaceutical companies were contacted for additional references or unpublished studies.

### 2.3. Data Collection

Two reviewers (X. Xiong and X. Yang) independently screened the titles, abstracts, and key words of each searched article for potentially eligible studies. The full-text articles were retrieved for further assessment if the information given suggests that the study (1) included old patients with essential hypertension, (2) compared KT formula with western medication in the presence or absence of cointerventions in both groups, (3) assessed one or more relevant clinical outcome measure such as morality and BP, and (4) used random allocation. The extracted information included study population; participant demographics and baseline characteristics; details of the intervention and control conditions; study methodology; outcome measures and main results. Any discrepancies were identified and resolved through discussion with a third author (J. Wang) if necessary.

### 2.4. Risk of Bias (Methodological Quality) Assessment

Two reviewers (X. Xiong and X. Yang) independently assessed the risk of bias for each trial according to the Cochrane Handbook for Systematic Reviews of Interventions version 5.1.0 [22]. The following items were assessed: random sequence generation (selection bias), allocation concealment (selection bias), blinding (performance bias and detection bias), incomplete outcome data (attrition bias), and selective outcome reporting (reporting bias). Based on Cochrane handbook, the risk of bias was categorized as low/unclear/high risk of bias. Trials which met all criteria were judged as having a low risk of bias, trials which met none of the criteria were judged as having a high risk of bias, and trials with insufficient information to judge were classified as unclear risk of bias. Disagreements were resolved by a third reviewer (J. Wang) when necessary.

### 2.5. Data Analysis

We performed meta-analyses using RevMan 5.1 software provided by Cochrane Collaboration for data analyses. Dichotomous data were expressed as risk ratio (RR) for binary outcomes and mean difference (MD) for continuous outcomes, with their 95% confidence intervals (CI), respectively. If required data were not reported, we requested data from corresponding author. We used fixed effects model unless there was evidence of heterogeneity. The statistical heterogeneity was examined with the *I* ^2^-test, where *I* ^2^ values of 50% or more were considered to be indicator of a substantial heterogeneity. In the absence of significant heterogeneity, we pooled data using a fixed-effect model (*I* ^2^ < 50%); otherwise we used random effects model (*I* ^2^ > 50%) [22].

## 3. Result

**Figure 1:**
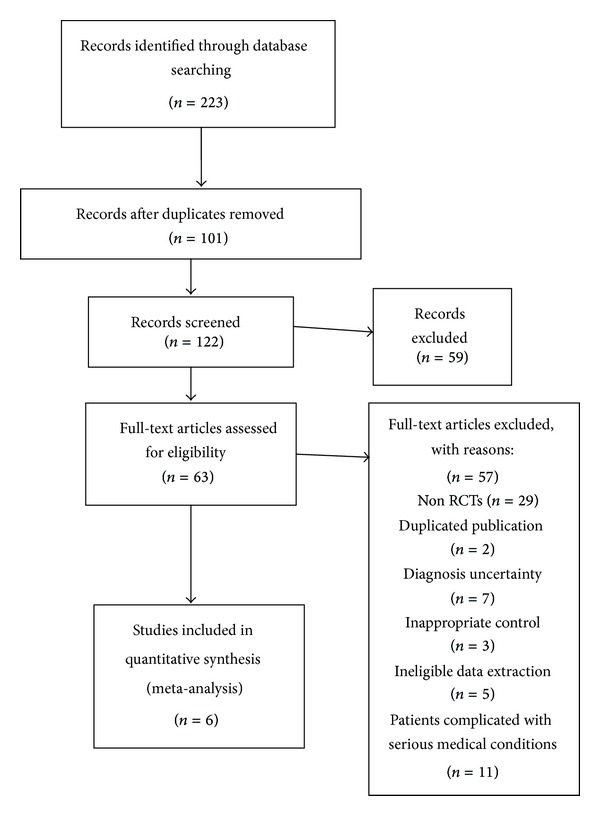
Flow Diagram of the literature searching and study selection.

A total of six trials [16–21], involving 527 participants with senile hypertension, were included in meta-analysis (see Table 1 and Figure 1). All 6 trials were conducted in China. The treatment duration ranged from 2 to 24 weeks. One trial [17] compared KT formula versus captopril. The other 5 trials compared KT formula plus western medications versus western medications alone. There was one placebo controlled study [16]. Only one trial [16] mentioned outcome measure of mortality and adverse events. All trials reported decreasing of BP. The compositions of different KT prescription were presented in Table 2.

Methodologically, all studies were at high risk of bias (see Table 3). One trial [16] described the method of randomization in detail, and the method was also appropriate. All the other studies did not report information on the allocation concealment. None were double blinded. The follow-up was recorded in one study [16]. No studies conducted intention-to-treat analysis.

### 3.1. Primary Outcomes

No trial adopted mortality as primary outcome. There was no report of the incidence of any heart event (e.g., acute myocardial infraction, severity arrhythmia, heart failure, and revascularization).

### 3.2. Secondary Outcomes

#### 3.2.1. KT Formula versus Western Medications

One trial [17] compared KT formula alone versus western medicine. There was no significant difference between the two groups in systolic blood pressure (MD: −3.70 (−11.47, 4.07); *P* = 0.35) and diastolic blood pressure (MD: −3.00 (−7.26, −1.26); *P* = 0.17) after 24 weeks of treatment (see Table 4).

#### 3.2.2. KT Formula Plus Western Medications versus Western Medications

A total of 5 trials [16, 18–21] compared the combination of modified KT formula plus antihypertensive drugs with antihypertensive drugs. Three trials [16, 19, 20] showed that there was a significant difference between treatment and control group in systolic blood pressure (MD: −8.69 (−12.35, −5.02); *P* < 0.00001) but no significant difference between treatment and control group in diastolic blood pressure (MD: −1.32 (−7.93, −5.30); *P* = 0.7) (see Table 5).

### 3.3. Other Outcomes (BL, ET, BUN, and Cr)

Two trials [16, 17] showed that after treatment, the level of blood lipid (BL) decreased significantly (*P* < 0.05) in KT formula group. Two trials [18–20] showed that after 2–8 weeks of treatment, the level of endothelin (ET) decreased significantly (*P* < 0.01) in KT formula group plus western medications group compared to western medications group. Furthermore, two trials [20, 21] showed that after 8 weeks of treatment, the level of blood urea nitrogen (BUN) and creatinine (Cr) decreased significantly (*P* < 0.05) in KT formula group plus western medications group compared to western medications group.

### 3.4. Adverse Effect

Only one trial mentioned the adverse effect in two groups such as palpitation, dizziness, and sleep [16]. These adverse effects disappeared after continuing to take medicine. The other five trials [17–21] reported no side effect in the KT formula group compared to antihypertensive drugs.

## 4. Discussion

In recent years, systematic reviews (SRs) and meta-analysis of CHM for treating hypertension have been conducted increasingly [23, 24]. To the best of our knowledge, this is the first systematic review and meta-analysis of RCTs for KT formula in treating senile hypertension. Our results showed that KT formula may be good to lower BP, especially for SBP. Based on the findings of meta-analyses, the preparation of KT formula used alone or combined with antihypertensive drugs may have some beneficial effects on the level of BL, ET, BUN, and Cr in old patients with essential hypertension. However, there were also some limitations of this review, which obstruct us to draw definite conclusion.

The most significant limitation is that all trials are at high risk of bias, which makes the findings less compelling. Except for one trial [16], none of the other trials reported the method of randomization, they only mentioned that “patients were randomized into two groups”, with no detailed information of randomization generation. Meanwhile, all trials claimed randomization, but they failed to provide enough information to judge whether the randomization procedures had been carried out properly. Only one placebo control was used [16], but none of the trials was of double-blind. In addition, no multicenter, large-scale RCTs were identified, and no dropouts and withdrawals were described. All the included trials used BP as outcome measure, but only half of the included trials evaluated the effectiveness with numerical values, which made the strengthening of evidence weak. It is recommended that future researchers should follow the basic guidelines for reporting clinical trials such as the Consolidated Standards of Reporting Trials (CONSORT) statement.

Several professional databases have been searched up to November 2013 including the Cochrane library, PubMed, CBM, CNKI, and VIP. After searching these databases, we found that all included trials were conducted and published in Chinese. The number of included trials is too small to conduct funnel plot analysis. So, the possibility of publication bias of KT formula for SH is unclear. In addition, there may be a limitation of missing some trials since we cannot search pertinent English databases like EMBASE.

Though none of the included trials reported severe adverse events possibly related to KT formula, we cannot draw firm conclusions about the safety of KT formula since seven trials did not report information on safety. The duration of treatment in most trials was 2 weeks or 8 weeks, so the potential toxin effect of KT formula for treatment of SH might only result from symptomatic changes (such as palpitation, dizziness, and sleep) and short treatment duration. The liver and kidney toxicity of Chinese medicines had drawn increasing attention to the concern of safety [25, 26]. We recommend that further researchers should focus on the monitoring and reporting of adverse events and long-term safety by designing a longer duration of treatment and a long-term follow-up including health-related quality of life [27, 28].

## 5. Conclusions

Our review suggested that compared with antihypertensive medications alone, KT combined with western medications was of more benefits for patients with SH. However, considering the strength of the evidence, more rigorously designed, randomized double-blind placebo controlled trials are required for assessing the effects of KT formula before recommended routinely. Nevertheless, some aspects must be specially considered in future including methodological improvement, adverse effects, and reporting clinically outcomes from long-term follow-up. All of this needs more well-designed, long-tern clinical trials.

## Figures and Tables

**Table 1:** Characteristics and methodological quality of included studies.

Study ID	Sample	Diagnosis standard	Intervention	Control	Course (week)	Outcome measure
Lu et al., 2007 [16]	90	JNC-7	KT formula plus indapamide	Placebo plus indapamide	24	BP; BL; adverse effects
Hu et al., 2001 [17]	84	CGPMHBP-1987	KT formula	Captopril	2	BP; BL
Huang et al., 2007 [18]	60	1999 WHO-ISH GMH	KT formula plus amlodipine basylate	Amlodipine basylate	2	BP; ET
Kong, 2003 [19]	60	1999 WHO-ISH GMH	KT formula plus plendil	Plendil	4	BP
Zhang and Li, 2006 [20]	136	1999 WHO-ISH GMH	KT pill plus cilazapril	Cilazapril	8	BP; ET; BUN; Cr
Zhang et al., 2008 [21]	97	CGPMHBP-2005	KT pill plus captopril	Captopril	8	BP; BUN; Cr

Abbreviations: KT: kidney-tonifying; WHO-ISH GMH, WHO-ISH guidelines for the management of hypertension; CGPMHBP: China Guidelines on Prevention and Management of High Blood Pressure; JNC-7: Seventh Report of the Joint National Committee on the Prevention, Detection, Evaluation, and Treatment of High Blood Pressure; BP: blood pressure; BL: blood lipid; ET: endothelin; BUN: blood urea nitrogen; Cr: creatinine.

**Table 2:** Assessment of risk of bias in included studies.

Study	Random sequence generation	Allocation concealment	Blinding	Incomplete outcome data	Selective reporting	Free of other biases	Summary assessments
Lu et al., 2007 [16]	Low risk	Unclear	Unclear	Unclear	Unclear	Low risk	High risk
Hu et al., 2001 [17]	Unclear	Unclear	Unclear	Unclear	Unclear	Unclear	High risk
Huang et al., 2007 [18]	Unclear	Unclear	Unclear	Unclear	Unclear	Unclear	High risk
Kong, 2003 [19]	Unclear	Unclear	Unclear	Unclear	Unclear	Unclear	High risk
Zhang and Li, 2006 [20]	Unclear	Unclear	Unclear	Unclear	Unclear	Unclear	High risk
Zhang et al., 2008 [21]	Unclear	Unclear	Unclear	Unclear	Unclear	Unclear	High risk

**Table 3:** Composition of different KT prescriptions.

Study ID	Preparation	Composition
Lu et al., 2007 [16]	Decoction	Cortex Eucommiae, Herba Taxilli, Fructus Ligustri Lucidi, Radix Astragali, Herba Epimedii, Rhizoma Polygonati, Rhizoma Alismatis, Radix Cyathulae, Rhizoma Chuanxiong, Radix Saposhnikoviae, Hirudo, Bombyx Batryticatus, and Rhizoma Gastrodiae (without details of dose). For kidney-yang deficiency, Radix Aconiti Lateralis Praeparata and Herba Cistanches were needed. For kidney-yin deficiency, Radix Rehmanniae Praeparata and Fructus Lycii were needed.

Hu et al., 2001 [17]	Decoction	Herba Taxilli 15 g, Herba Epimedii 15 g, Fructus Ligustri Lucidi 15 g, Radix et Rhizoma Salviae Miltiorrhizae 30 g, Rhizoma Chuanxiong 12 g, Radix et Rhizoma Notoginseng 5 g, Radix Puerariae Lobatae 30 g, Rhizoma Alismatis 12 g, and Poria 12 g. For kidney-yin deficiency, Radix Rehmanniae Praeparata and Fructus Lycii were needed. For kidney-yang deficiency, Radix Aconiti Lateralis Praeparata, Cortex Cinnamomi, and Herba Cistanches were needed. For yin deficiency with yang hyperactivity, Radix Paeoniae Alba, and Rhizoma Gastrodiae were needed.

Huang et al., 2007 [18]	Decoction	Cortex Eucommiae 15 g, Herba Taxilli 15 g, Pheretima 15 g, Radix et Rhizoma Salviae Miltiorrhizae 15 g, Herba Leonuri 15 g, Fructus Corni 15 g, and Cortex Moutan 10 g.

Kong, 2003 [19]	Decoction	Radix Rehmanniae 15 g, Radix Rehmanniae Praeparata 15 g, Polyporus, Poria 15 g, Rhizoma Dioscoreae 25 g, Fructus Corni, Rhizoma Alismatis 10 g, Cortex Moutan 10 g, Radix Achyranthis Bidentatae 10 g, Pheretima 10 g, Semen Plantaginis 30 g, and Radix et Rhizoma Salviae Miltiorrhizae 30 g. For yin deficiency with yang hyperactivity, Rhizoma Gastrodiae 15 g and Concha Margaritiferae Usta 30 g were needed. For deficiency of both yin and yang, Cortex Cinnamomi 3 g and Radix Aconiti Lateralis Praeparata 10 g were needed.

Zhang and Li, 2006 [20]	Pill	Rhizoma Alismatis, Rhizoma Dioscoreae, Cortex Moutan, Fructus Corni, Poria, and Radix Rehmanniae Recens (without details of dose).

Zhang et al., 2008 [21]	Pill	Rhizoma Alismatis, Rhizoma Dioscoreae, Cortex Moutan, Fructus Corni, Poria, and Radix Rehmanniae Recens (without details of dose).

Abbreviations: KT: kidney-tonifying.

**Table 4:** Analyses of systolic blood pressure.

Trials		MD [95% CI]	*P* value
*KT versus antihypertensive drugs *			
KT formulae versus captopril	1	−3.70 [−11.47, 4.07]	0.35

Meta-analysis	1	−3.70 [−11.47, 4.07]	0.35

*KT plus antihypertensive drugs versus antihypertensive drugs *			
KT formula plus felodipine versus felodipine	1	−10.03 [−15.53, −4.53]	0.0004
KT formula plus indapamide versus indapamide	1	−2.10 [−9.85, 5.65]	0.60
KT pill plus cilazapril versus cilazapril	1	−10.00 [−11.79, −8.21]	<0.00001

Meta-analysis	3	−8.69 [−12.35, −5.02]	<0.00001

Abbreviations: KT: kidney-tonifying.

**Table 5:** Analyses of diastolic blood pressure.

Trials		MD [95% CI]	*P* value
*KT versus antihypertensive drugs *			
KT formulae versus captopril	1	−3.00 [−7.26, 1.26]	0.17

Meta-analysis	1	−3.00 [−7.26, 1.26]	0.17

*KT plus antihypertensive drugs versus antihypertensive drugs *			
KT formula plus felodipine versus felodipine	1	−0.78 [−2.61, 4.17]	0.65
KT formula plus indapamide versus indapamide	1	2.80 [−1.30, 6.90]	0.18
KT pill plus cilazapril versus cilazapril	1	−7.00 [−8.00, −5.20]	<0.00001

Meta-analysis	3	−1.32 [−7.93, 5.30]	0.70

Abbreviations: KT: kidney-tonifying.
